# A Historic Review of the Three-Century Journey of the Circulus Arteriosus Cerebri: Then and Now

**DOI:** 10.7759/cureus.91218

**Published:** 2025-08-29

**Authors:** Jessica C Garlick, Graham Louw, Kentse Mpolokeng

**Affiliations:** 1 Department of Human Biology, Faculty of Health Science, University of Cape Town, Cape Town, ZAF

**Keywords:** cerebral arterial circle, circle of willis variations, circulus arteriosus cerebri variations, classification systems, de cerebri anatome

## Abstract

The *circulus arteriosus cerebri* (CAC) has been a subject of anatomical study for over three centuries and remains a structure of anatomical and clinical interest due to its highly variable nature. This review highlights how differences in nomenclature, classification systems, sample sizes, and methodologies have contributed to the variability observed in the literature. These factors complicate direct comparisons across studies and may influence the interpretation of clinically relevant data. Of note, such anatomical variations can directly impact clinical diagnosis, surgical planning, and patient outcomes, which highlights the need for accurate and consistent classification of these variations. A historical perspective of the study of the CAC illustrates how evolving technologies and terminology have shaped the current understanding of this structure. Future research may benefit from clearly defined variation criteria, detailed methodological reporting, and careful consideration as to which data are appropriate for comparison. These practices could support more consistent and interpretable findings in the continued study of the CAC and its anatomical variations.

## Introduction and background

Historical background

The *circulus arteriosus cerebri* (CAC) is an anastomosis of nine arteries at the base of the brain, known for its highly variable pattern. The CAC provides a protective mechanism by enabling the rapid compensation of cerebral blood flow when perfusion is reduced through one of its components or closely related arteries [1,2]. This compensatory function, referred to as collateral blood supply, helps mitigate the impact of ischemic events by maintaining perfusion to affected brain regions [3,4]. The CAC is also believed to mitigate the adverse effects of fluctuations in cerebral blood flow by maintaining consistent cerebral perfusion. Marked fluctuations in blood flow through one or more CAC arteries can be redistributed within the circle, thereby reducing hemodynamic pressure within individual component arteries and their downstream branches [1,3]. 

The considerable variation within the CAC has led to extensive research into it. Currently, several factors make direct comparisons of the literature complicated, and these factors contribute to the wide range of frequencies reported for variations [5]. These factors could impact the reliability of clinically significant diagnoses and procedures related to the CAC and include differences in nomenclature [6,7], the classification system used [8], sample size [8,9], cohort selection, and methodology used [5,8-10]. To gain a comprehensive understanding of these factors, it is essential to examine the long history of research on CAC. This review explores the historical and scientific development of our understanding of the CAC since its formal introduction to the scientific world in 1664 by Dr. Thomas Willis, through to the dynamic world of anatomy today, in the 21st century.

This has been an exciting three-century journey that continues to unlock deeper knowledge of the brain and its blood supply using different technologies, techniques, and a living language that is used to describe the CAC arteries. Although the CAC was formally identified in 1664, knowledge of this arterial anastomosis had existed for an extensive period of time, dating back to the Greco-Roman Antiquity starting in approximately the eighth century BC [11]. Some scholars, like Johann Vesling and Johann Jakob Wepfer von Schaffhausen [11], provided partial descriptions of the CAC before Willis. However, it was Willis who first offered a complete and detailed description of this arterial system and determined its function and significance in his publication of De Cerebri Anatome in 1664 [10,12,13]. Additionally, with the help of Sir Christopher Wren as his illustrator, Willis was the first to provide a complete illustration of the CAC [11]. Research into the CAC continued, and a century later, Albrecht von Haller coined the phrase “the Circle of Willis” for the CAC [11], a name that is in use today, specifically in the clinical setting.

This review aims to investigate the historical development of the CAC with a focus on identifying and understanding the factors that contribute to inconsistencies and challenges in comparing findings across the existing literature. This review covers foundational anatomical studies, evolving methodological approaches, and variations in classification systems documented over time. This work draws on both traditional and current literature to highlight the role of historical context in shaping the ongoing research of the CAC.

## Review

Anatomical variability

**Figure 1:**
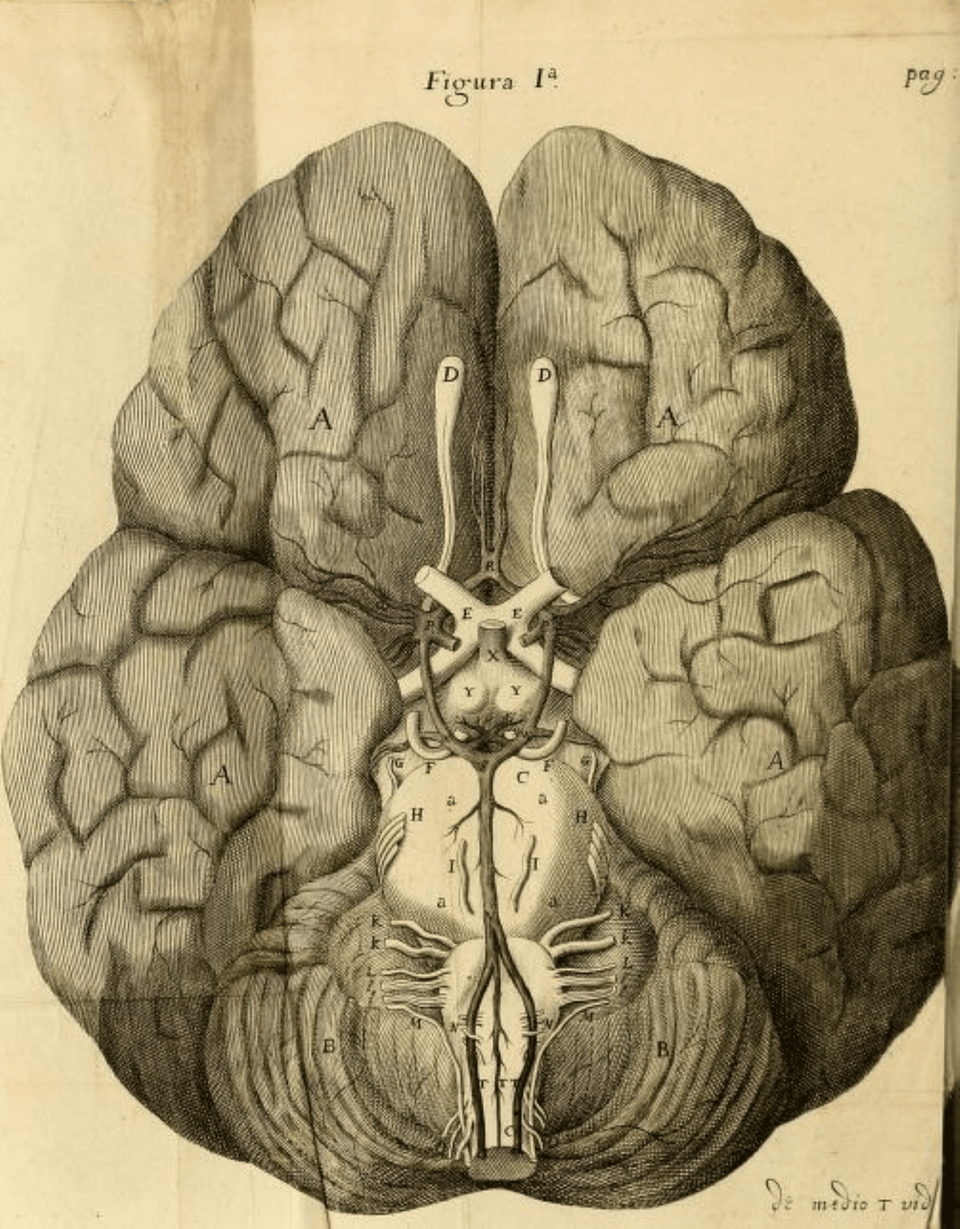
An illustration of the CAC on the base of the brain From ‘De Cerebri Anatome’ (page 25), published by Thomas Willis in 1664 and illustrated by his associate Christopher Wren. Image reproduced from the Wellcome Collection under Creative Commons Attribution 4.0 International Licence (CC BY 4.0) [15]. Note: The features labelled with letters of the alphabet cover a range of anatomical structures as identified by the original author CAC: *circulus arteriosus cerebri*

Dr. Willis described the conventional configuration of the CAC in his 1664 publication (Figure 1), and this description still stands today. The conventional CAC configuration is a complete, symmetrical polygonal ring formed by four main arteries: the left and right internal carotid arteries (ICAs) and vertebral arteries (VAs) [2,6,14]. Each ICA gives rise to an anterior cerebral artery (ACA) and a middle cerebral artery (MCA) on the ipsilateral side. The ACAs are connected by the anterior communicating artery (AcoA), while the VAs fuse posteriorly to form the basilar artery (BA), which bifurcates into the left and right posterior cerebral arteries (PCAs). The posterior communicating artery (PcoA), typically branches from the ICA, links the PCA to the ipsilateral ICA, completing the ring [6].

Studies consistently report that fewer than 50% of individuals exhibit the conventional anatomical configuration of the CAC because of the high prevalence of anatomical variations [6,16]. Common variations include hypoplasia of one or more arteries [17,18], aplasia or absence of arteries [17-19], arterial fenestrations [20], and the presence of accessory arteries, such as duplications or triplications [6,18]. In some cases, arteries originate from atypical sites, deviating from the conventional branching pattern [21]. These variations can have important clinical implications, as they may alter cerebral haemodynamics [22], complicate the interpretation of diagnostic imaging, and impact surgical or endovascular planning and outcomes [22-24].

Classification systems

Given the high variability in the CAC, several researchers have since developed classification systems, absent from Willis’s original description, to facilitate clearer comparisons across studies and to advance understanding of this complex arterial network. Over the past three centuries, the classification of the CAC has evolved from basic anatomical descriptions of individual variations to more detailed classification systems. Despite numerous frameworks proposed in the literature, there is currently no standardized system for recording and reporting anatomical variations of the CAC. As a result, researchers often adopt different classification schemes, with 46.88% even developing their own according to the meta-analysis conducted by Jones et al. [5].

Others modified or used a pre-existing system, such as the one developed by Lazorthes et al. [25], which classified variations into 22 types according to artery diameter. The system developed by Chen et al. [26], which divided variations into 15 anterior and 15 posterior circulation variations, and Ayre et al. [27], which separated variations into 82 variants (types) within five continuous groups. A comparison of the nomenclature used by these three classification systems is summarized in Table 1. Ayre et al. [27] proposed a system that classifies both recorded and unrecorded variations, benefiting past and future studies. In contrast, the more established systems by Lazorthes et al. [25] and Chen et al. [26] lack this flexibility. Notably, Ayre et al. [27] distinguish between aplastic and hypoplastic arteries, allowing for two combination options, whereas the other systems do not differentiate between these variations.

**Table 1:** Comparison of the nomenclature used in three classification systems of the CAC The 22 variation types described by Lazorthes et al. [25] are compared to the 15 anterior and 15 posterior circulation variants identified by Chen et al. [26], and to the 82 types, classified into five groups, by Ayre et al. [27]. “No match” indicates that there was not a type in the relevant classification system that corresponded to the other two classification systems. Not all the potential variations are included in the table, but only those where potential comparisons could be made. CAC: *circulus arteriosus cerebri*; AcoA: anterior communicating artery; A1: pre-communicating segment of the anterior cerebral artery; PcoA: posterior communicating artery; P1: pre-communicating segment of the posterior cerebral artery; MedACA: median anterior cerebral artery; PCA: posterior cerebral artery

Standard configuration and variations	Nomenclature used by classification systems		
	Lazorthes et al., 1979	Chen et al., 2004	Ayre et al., 2021
Standard configuration	Type 1	No match	No match
Hypoplasia			
AcoA	Type 3	Anterior type G	Group 1: type 1
Unilateral A1	Type 8	No match	Group 1: type 2
Unilateral PcoA	Type 4	No match	Group 1: type 3
Bilateral PcoA	Type 6	Posterior type E (non-patent)	Group 1: type 11
Unilateral P1	Type 9	No match	Group 1: type 4
Bilateral P1	Type 10	Posterior type C (both patent), posterior I (non-patent)	Group 1: type 13
Aplasia			
AcoA (no connection)	No match	Anterior type G	Group 2: type 25
AcoA (long fusion)	No match	Anterior type E	No match
AcoA (short fusion)	No match	Anterior type D	No match
Unilateral A1	No match	Anterior type H	Group 2: type 26
Unilateral PcoA	No match	Posterior type D	Group 2: type 27
Bilateral PcoA	No match	Posterior type E	Group 2: type 31
Unilateral P1	No match	No match	Group 2: type 28
Bilateral P1	No match	Posterior type I	No match
Multiplications			
Triple AcoA	No match	Anterior type B	Group 4: type 51
Duplicated AcoA (Parallel)	No match	Anterior type B	Group 4: type 42
Duplicated (H) AcoA	No match	Anterior type B	No match
Duplicated (V or Y) AcoA	No match	Anterior type B	Group 5: type 70
Plexiform AcoA	No match	Anterior type B	No match
MedACA	No match	Anterior type C	Group 4: type 52
Unilateral A1	No match	No match	Group 4: type 59
Duplicated PCA	No match	No match	Group 4: type 62 (unilateral)
Combined variations			
Unilateral hypoplasia of PcoA and AcoA	Type 5	No match	Group 1: type 5, group 2: type 29 (absent)
Bilateral hypoplasia of PcoAs and AcoA	Type 7	No match	Group 1: type 15, group 2: type 32 (absent)
Hypoplasia of the P1 and contralateral A1	Type 11	No match	Group 1: type 10
Hypoplasia of the P1 and ipsilateral A1	Type 12	No match	Group 1: type 9
Bilateral hypoplasia of P1s and A1s	Type 13	No match	No match
Hypoplasia of A1 and contralateral PcoA	Type 14	No match	Group 1: type 8
Hypoplasia of AcoA and P1	Type 15	No match	Group 1: type 6
Hypoplasia of the PcoA, ipsilateral A1, and AcoA	Type 16	No match	No match
Hypoplasia of the PcoA and contralateral P1	Type 17	Posterior type B (both patent), posterior type F (P1 non-patent), posterior type G (PcoA non-patent), posterior type H (both non-patent)	Group 1: type 12
Bilateral hypoplasia of the PcoAs and A1	Type 18	No match	Group 1: type 18, group 2: type 33 (absent)
Hypoplasia of the PcoA, AcoA, and contralateral P1	Type 19	No match	Group 1: type 16
Hypoplasia of the P1, contralateral PcoA, and ipsilateral A1	Type 20 (left PcoA), type 22 (right PcoA)	No match	Group 1: type 19
Bilateral hypoplasia of the P1s and AcoA	Type 21	No match	Group 1: type 17

Table 1 illustrates how varying nomenclature across classification systems may be challenging to compare between studies owing to perceived ambiguities [27,28]. This is especially true for early-career researchers and postgraduate students. The authors of this review believe Table 1 may assist others in comparing their findings with existing literature. Inconsistent terminology can impact the interpretation of these systems, as similar variations may be classified differently or described using interchangeable terms. Furthermore, some studies, like Papantchev et al. [23], specify the side of unilateral variations, while others, such as Riggs et al. [29], do not, potentially influencing perceived haemodynamic flow or limiting cross-study comparisons. Ideally, a consensus should be established to allow for more consistent comparisons. Jones et al. [5] suggest creating a database to assist with the development of a universal classification for the CAC. However, this may prove challenging as many researchers often prefer systems they are familiar with, those that suit their study needs, or may choose not to use a system at all. Regardless, it is recommended that authors clearly state the classification system and definitions used for reporting variations to enable others to more reliably compare the results to their own data. It is also valuable if researchers think critically when comparing studies that use different classification systems to avoid grouping different variations in their comparisons.

In the authors’ research, the classification system by Ayre et al. [27] was used because it distinguished between hypoplastic and aplastic arteries and allowed for combinations of variations in both the anterior and posterior circulations. Its grouped arrangement also enabled the inclusion of variations not captured in the pre-existing types described by Ayre et al. [27]. While this system may benefit future studies, comparing results across different classification systems remains difficult, as equivalent variation types are not always present. However, as the Ayre et al. [27] system is relatively new, broader adoption or detailed reporting of variations by other researchers would enhance comparability.

Terminology and standardization challenges

The terminology used to describe CAC variations has evolved, making it difficult to compare studies, especially when comparing more recent findings with historic results, including the original descriptions by Willis. For example, early researchers described “very small” or “abnormally small” arteries [21], which we now refer to as hypoplastic arteries. However, the exact definition of ‘hypoplasia’ has also changed over time, highlighting the need for authors to specify their chosen definition. Definitions vary widely. Iqbal [6] and Kamath [12] defined hypoplastic arteries as those with a diameter smaller than 0.5 mm or 1 mm, depending on the artery. Others, including Chen et al. [25], Gaigalaite et al. [13], and Machasio et al. [30], used a threshold of less than 0.8 mm. Lazzaro et al. [22] and Zimelewicz Oberman et al. [31] defined hypoplasia as an artery with a diameter less than 50% of the largest A1 segment. Meanwhile, 56.25% of studies [5], such as those conducted by Eftekhar et al. [32], Cilliers and Page [33], Saikia et al. [17], and Kapoor et al. [18], used a fixed threshold of less than 1 mm, irrespective of the artery being analyzed. Notably, 18.75% of studies did not define hypoplasia at all [5], complicating cross-study comparisons. Without a clearly stated definition, such studies cannot be reliably used in comparative analyses.

In 2000, Hoksbergen et al. [3] concluded that the threshold diameter of the AcoAs and PcoAs ranges from 0.4 mm to 0.6 mm. Future studies should build on this to determine whether a universal threshold exists across all arteries or if separate values are needed for the communicating arteries, potentially leading to a revised and more standardized definition.

A related issue is the interchangeable use of ‘aplasia’ and ‘hypoplasia’. Aplasia typically refers to an absent artery, has a diameter of zero, or, in the case of radiological studies, shows no blood flow (is non-patent). This confusion may stem from the incorporation of radiologic methods into CAC research. Comparisons between studies that do not distinguish between aplasia and hypoplasia and those that do may be unreliable and should be interpreted with caution. Differences in the use of nomenclature are not limited to hypoplasia and aplasia. Several other variations are also affected, contributing to a wider range of measurements of CAC arteries or prevalence of variations being reported [6,7]. For instance, terms like azygos ACA and median ACA (MedACA) are sometimes used interchangeably [18,34], even though these are different variations. This confusion potentially arose because both variations develop from the persistence of the median artery of the corpus callosum [35].

The language used to describe the CAC has gradually evolved alongside our growing understanding of its anatomy. While this progression reflects scientific advancement, it also presents challenges for modern researchers, particularly when comparing studies that use different or outdated terminology. To address this, it is essential that authors clearly define the anatomical variations they report. Although universal standardisation of all variation definitions may be unrealistic because of the evolving nature of anatomical knowledge, researchers must demonstrate a clear understanding of the variants they describe and provide concise, explicit definitions. Efforts to promote greater consistency include international initiatives such as the Federative International Programme for Anatomical Terminology (FIPAT) [36] and the publication of *Terminologia Anatomica* [37], both of which aim to standardize anatomical language across disciplines and regions. Nevertheless, terms like ‘hypoplasia’ continue to be defined inconsistently between studies, highlighting the ongoing need for clarity and transparency in CAC research.

Methodological approaches

In Dr. Willis’s time, macroscopic dissection was the only method available to study the CAC. Since then, advances in technology, particularly radiographic imaging, have expanded the range of techniques used. Each method provides valuable insights about the CAC, but varies in reliability, and its limitations should be clearly acknowledged.

Dissection studies, although foundational, are not uniform. Some use embalmed brains, while others rely on unembalmed brains. Additionally, embalming solutions and techniques vary widely, potentially influencing arterial dimensions. There is an ongoing debate on whether embalming increases or decreases vessel dimensions. For example, Balta et al. [38] took CT scans of blood vessels before and after the embalming process and showed that embalming fluid pressure caused the diameter of arteries to increase, fixing them in an enlarged state [38]. Saikia et al. [17] similarly noted that small arteries may increase in diameter over time, potentially causing hypoplastic arteries to appear average-sized [9, 17]. In contrast, others argue that artery diameters may decrease post-mortem due to loss of blood pressure and arterial muscle decay [7,9]. Formaldehyde, commonly used in South African medical schools, may increase arterial wall rigidity and shrinkage [39], potentially resulting in average-diameter arteries being misclassified as hypoplastic.

We hypothesize that acidic embalming solutions may cause arterial constriction, a view supported by Hoksenbergen et al. [3] and Van Eijk et al. [35]. If such constriction occurs, then arterial diameters may be smaller than in vivo. However, the opposing effects of embalming fluid pressure and chemical constriction may balance each other out. Comparative studies of embalmed versus unembalmed brains, or pilot studies examining the specific embalming methods, are needed to clarify these effects. For these reasons, some researchers prefer to use unembalmed, refrigerated samples to avoid embalming artifacts [39]. However, freezing and thawing may also affect arterial size and preservation quality, which could cause varying results between samples [17]. Despite these concerns, embalmed samples remain more accessible and practical in educational settings owing to their firmness and resistance to breakage [16]. In fact, some studies embalm unembalmed samples before dissection for this reason [10,16,18]. 

Measurement methods for arterial diameters in dissection studies also vary, contributing further to inconsistent results [10]. Furthermore, random sampling is usually not possible with dissection-based studies owing to the nature of the research. The non-random sampling of dissection studies may result in bias (e.g., older age groups dominate the samples) and not fully reflect the anatomical variation found in the wider population. All these factors must be considered when comparing and interpreting data from the literature. In more recent years, radiographic techniques, such as multi-slice CT angiography (CTA) [40], digital subtraction angiography (DSA) [41], and time-of-flight magnetic resonance imaging (3D-TOF-MRA) [17, 26], have gained popularity owing to their ability to study living populations [2,17,42]. Rapid advances in imaging technology enable researchers to adopt cutting-edge methods, some of which are non-invasive [42].

However, these techniques differ in sensitivity, spatial resolution, specificity, and accuracy in diagnosing different conditions. Some, such as 3D-TOF-MRA and CTA, rely on blood flow volume and direction, which may lead to very small arteries with slow or turbulent flow being classified as aplastic [17]. This can underestimate the completeness of the CAC, contrasting with dissection studies where non-patent arteries may still be visible [2,31]. This anatomical versus physiological completeness highlights the importance of patency testing in dissection studies [18].

Direct comparisons between dissection and radiographic studies are challenging since dissections measure the external diameters without blood pressure [10], while imaging studies measure the internal diameter under blood pressure or physiological conditions [9,10,32]. Despite this, a meta-analysis by Jones et al. [5] found no statistically significant differences between these two approaches, although no consistent pattern or correlation was observed either.

Ultimately, each methodology has its own set of strengths and limitations, influenced by factors such as funding, technological resources available, researcher preference, and institutional or geographic context. This review highlights the methodological diversity in CAC research and its impact on comparability. Researchers must acknowledge these differences, carefully select comparable studies, and transparently report their own methodological limitations.

**Table 2:** Summary of key differences between dissection and radiographic studies of the CAC CAC: *circulus arteriosus cerebri*

Feature	Dissection Studies	Radiographic Studies
Population	Cadaveric/body donor samples, usually embalmed, but also unembalmed [6,9,12,18]	Living populations, often hospital or clinic patients [2,8,26,41]
Condition of arteries	Arteries may be collapsed because of a lack of blood pressure, or have post-mortem alterations [6,12,19,38]	Assessed under physiological pressure; arteries assessed in functioning state [1,4,26,31]
Measurement type	External arterial diameter or length [16,18]	Internal arterial diameter or length [8,26,41]
Effect of embalming	May increase or decrease arterial size depending on embalming fluid solution, pressure, and fixation method [12,17,38]	Not applicable
Effect of freezing/thawing (unembalmed samples)	Possible impact on artery dimensions [6,9,18]	Not applicable
Artery visibility	All vessels visible structurally (unless aplastic) regardless of patency [6,9,12,19]	Limited by flow: hypoplastic arteries may not be visualized if flow is slow or absent [4,8,26,31,41]
Classification limitations	Risk of misclassifying average arteries as hypoplastic (shrinkage), or vice versa (expansion) [6,12,38]	Risk of classifying present arteries as aplastic due to slow or turbulent flow [8,26,31,41]
Common techniques used	Callipers, high-resolution photographs, or digital tools (e.g., Figi software) [6,9,18]	CTA, DSA with advanced image reconstruction, MRA (e.g., 3D-TOF-MRA) [8,26,31,41,42]
Invasiveness	Invasive and destructive [6,12]	Often non-invasive (if no contrast material, e.g., MRA) [26,41,42]
Accessibility	Requires access to body donors (cadaver samples) and dissection facilities [18,39]	Requires advanced imaging equipment and clinical access [8,41]
Common Use Cases	Anatomical education, post-mortem analysis, structural mapping	Clinical diagnosis, in vivo anatomical research, population studies [2,4]
Advantages/Strengths	Direct structural visualization, valuable for morphological classification, visualisation of non-patent arteries [6,12,38]	Enables study of living populations, non-invasive, allows for functional, in vivo analysis; potential for large-scale data collection and comparison [26,31,41]
Disadvantages/Limitations	Variable preservation methods, fixation artifacts (shrinkage, post-mortem changes, preservation technique variability) [6,12,18]	Image resolution limits, dependence on flow, possible under-visualisation of small vessels, potential imaging artifacts [31,41]
Level of detail	Detailed gross morphology (visibility limited by dissector’s skill level), useful for variant classification [12,19]	High resolution imaging enables 3D reconstructions and prevalence mapping [26,31,41]
Trend in use	More common in earlier research, but still valuable in anatomical literature	Increasingly used due to advancements in imaging and access to large population cohorts

Sample size and cohort selection

Over the past three centuries of CAC research, sample sizes have varied considerably between studies. Many studies report small sample sizes, which may not adequately represent the general population. The frequency of CAC variations observed may differ between similar studies if the sample sizes differ significantly [9]. For instance, sample sizes in dissection studies, excluding case reports, ranged from as few as 10 samples [2] to as many as 1000 samples [18]. A known limitation of dissection studies is their generally smaller sample size compared to radiological studies, mainly due to the availability of samples. As with most study designs, larger samples, especially those drawn at random, are more likely to be representative of the broader population. Moreover, studies with large sample sizes have a greater chance of recording uncommon or so-called ‘rare’ variations [9], and they yield more realistic estimates of variation frequency. In dissection halls, body donors may not capture the diversity of the general population. These bodies are donated for educational and research purposes, including teaching medical students through dissection or professional training workshops. Consequently, the donor pool may be skewed demographically or clinically.

Another factor contributing to the variability in reported CAC patterns is the inclusion of brains with neurovascular diseases or conditions that could potentially alter vascular morphology. The conclusions made from such samples may be influenced by the underlying pathology [19,39,43,44]. For example, Arjal et al. [40] noted a potential selection bias when using radiographic images of brains from patients with cerebrovascular complaints. In many dissection studies, especially those without access to donor medical records, only brains with visible signs of pathology could be excluded. Ideally, studies should use brains with no pathologies that may have direct or indirect influences on the morphology of the CAC.

Despite these limitations, small-sample studies, including case reports, remain valuable and deserve publication. However, researchers must acknowledge these limitations when interpreting their own findings or comparing them to existing data. Furthermore, examining CAC patterns in pathological brains may offer valuable insights for studies focusing on specific diseases.

Geographical and demographic differences

As awareness and knowledge of the CAC have grown, research into its variations has expanded globally. In addition to age and sex, some studies have investigated associations between CAC variations and geographic location. Population-based differences in CAC configurations have been reported by Jones et al. [5], De Silva et al. [6], and Kornieieva et al. [45]. However, Eftekhar et al. [31] found no statistically significant difference between the populations compared in their study, an outcome that may have been influenced by the classification system used. De Silva et al. [6] further noted that geographical differences in the CAC include both intra- and inter-population variation, likely influenced by genetic, environmental, or combined factors [6,10]. Other factors that may have contributed include sex, age, socioeconomic status, health status, and environmental factors.

Such population-specific anatomical differences may contribute to variation in study findings across regions [5,10,32], including the differences observed in age and sex with artery dimensions. As such, increased region-specific research, including in underrepresented countries like South Africa, could enhance our understanding of CAC variations. Given the presence of intra-population variation [10], each province, state, or region within a country may reveal meaningful differences in the prevalence of variations. Future studies might also investigate the effect of environmental factors on the pattern and dimensions of the CAC.

## Conclusions

Our knowledge of the CAC has grown substantially thanks to the accumulated efforts of researchers over the past three centuries, including Dr. Willis and Sir Christopher Wren, who first illustrated the conventional CAC configuration in 1664. Since then, technological and methodological advancements, as well as the evolution of anatomical terminology, have contributed significantly to our understanding. While further changes are inevitable, it remains essential that researchers clearly define the variations they observe, communicate their descriptions accurately, and acknowledge the limitations of their study designs. These limitations may include sample size, the demographic profile of the cohort, or limitations associated with the chosen methodology. As scientists, we have a responsibility to be transparent about the limitations of our studies and to describe anatomical variations with enough detail to ensure they are fully understood by others. Given that variations in the CAC may alter collateral blood flow, influence radiological accuracy, and affect neurosurgical or endovascular procedures, in-depth and consistent reporting is important. We should also exercise caution when comparing data, ensuring that comparisons are made only between studies that are methodologically sound and appropriately aligned. Being mindful of the aforementioned factors while researching the CAC will contribute to the continuous development of understanding the CAC and its variations.
